# Extracellular Alpha-Synuclein Oligomers Induce Parkin *S*-Nitrosylation: Relevance to Sporadic Parkinson’s Disease Etiopathology

**DOI:** 10.1007/s12035-018-1082-0

**Published:** 2018-04-21

**Authors:** Anna Wilkaniec, Anna M. Lenkiewicz, Grzegorz A. Czapski, Henryk M. Jęśko, Wojciech Hilgier, Robert Brodzik, Magdalena Gąssowska-Dobrowolska, Carsten Culmsee, Agata Adamczyk

**Affiliations:** 1 Department of Cellular Signalling, Mossakowski Medical Research Centre, Polish Academy of Sciences, Pawińskiego 5 Street, 02-106 Warsaw, Poland; 2 Department of Neurotoxicology, Mossakowski Medical Research Centre, Polish Academy of Sciences, Pawińskiego 5 Street, 02-106 Warsaw, Poland; 3 BLIRT S.A., Trzy Lipy 3/1.38, 80-172 Gdańsk, Poland; 4 Institute of Pharmacology and Clinical Pharmacy, University of Marburg, 35043 Marburg, Germany

**Keywords:** Alpha-synuclein, Parkin, Parkinson’s disease, *S*-nitrosylation

## Abstract

α-Synuclein (ASN) and parkin, a multifunctional E3 ubiquitin ligase, are two proteins that are associated with the pathophysiology of Parkinson’s disease (PD). Excessive release of ASN, its oligomerization, aggregation, and deposition in the cytoplasm contribute to neuronal injury and cell death through oxidative-nitrosative stress induction, mitochondrial impairment, and synaptic dysfunction. In contrast, overexpression of parkin provides protection against cellular stresses and prevents dopaminergic neural cell loss in several animal models of PD. However, the influence of ASN on the function of parkin is largely unknown. Therefore, the aim of this study was to investigate the effect of extracellular ASN oligomers on parkin expression, *S*-nitrosylation, as well as its activity. For these investigations, we used rat pheochromocytoma (PC12) cell line treated with exogenous oligomeric ASN as well as PC12 cells with parkin overexpression and parkin knock-down. The experiments were performed using spectrophotometric, spectrofluorometric, and immunochemical methods. We found that exogenous ASN oligomers induce oxidative/nitrosative stress leading to parkin *S*-nitrosylation. Moreover, this posttranslational modification induced the elevation of parkin autoubiquitination and degradation of the protein. The decreased parkin levels resulted in significant cell death, whereas parkin overexpression protected against toxicity induced by extracellular ASN oligomers. We conclude that lowering parkin levels by extracellular ASN may significantly contribute to the propagation of neurodegeneration in PD pathology through accumulation of defective proteins as a consequence of parkin degradation.

**Electronic supplementary material:**

The online version of this article (10.1007/s12035-018-1082-0) contains supplementary material, which is available to authorized users.

## Introduction

Parkin and alpha-synuclein (ASN) are two major proteins associated with the pathophysiology of Parkinson’s disease (PD). Recessive mutations in the autosomal parkin gene *PARK2* are the most common causes of familial early onset PD, while dominant mutations in the autosomal ASN gene cause late onset PD [1]. In sporadic PD, ASN accumulates into cytosolic inclusions called Lewy bodies (LBs) [2]. Previous investigations revealed that the toxicity of overproduced and aggregated ASN is mainly attributed to the formation of “toxic ASN oligomers.” Those heterogeneous and till now poorly characterized group of ASN conformers may negatively impact cells by inducing oxidative-nitrosative stress, mitochondrial alterations, endoplasmic reticulum stress, proteasomal defects, synaptic dysfunction, and neuroinflammation [3–5]. A growing body of evidence further suggests that ASN may be able to self-propagate between neurons, in a prion-like manner, which may play a pivotal role in PD pathology. ASN may also act as a trigger of neurodegenerative processes associated with oligomerization of other amyloidogenic proteins, including amyloid-beta peptide (Aβ) [6] or through damage of protein degradation systems [7, 8]. On the contrary, the role of parkin in sporadic PD is explained mainly through functional inactivation due to nitrosative and oxidative stress [9–12] or altered dopamine metabolism [13]. This seems to be particularly significant to PD pathogenesis, since the major function of parkin as an E3-ubiquitin ligase is involved in ensuring the quality control of protein conformation and mitochondrial function [14–17].

Despite the lack of a genetic link between parkin and ASN, pathological interaction between ASN and parkin in sporadic PD has emerged as an important trigger of neurodegenerative processes [1]. Based on the genetic evidence and the established role of parkin as a ubiquitin ligase, the scientific interest was initially focused on investigating the link of parkin alterations and proteasomal dysfunction to ASN accumulation [7, 18]. However, it was demonstrated that ubiquitin-proteasome system (UPS) contributes to ASN turnover mainly in physiological conditions and only the glycosylated form of ASN is a substrate for parkin’s ubiquitin ligase activity in human brain tissue [18–20]. Further, subsequent studies showed that proteasomal degradation of ASN does not require the ubiquitination of this protein [21]. Interestingly, in pathological conditions, the increased intracellular ASN burden is recruited mainly into the autophagy-lysosomal pathway instead of UPS [20]. These observations indicated that aberrant ASN is less likely the direct substrate for parkin E3 ligase activity, which corresponds to studies showing that loss of parkin function (via mutations) is usually not associated with LBs [22]. Intriguingly, one recent publication demonstrated that neuroprotective properties of parkin activation are mediated by autophagic degradation of ASN [23]. Parkin also reduced the level of phosphorylated ASN in immortalized dopaminergic cells and attenuated ASN-induced glia activation [24]. Moreover, when parkin is down-regulated, it induces increased ASN secretion into the blood [23]. Since ASN oligomers display prion-like properties, including entry into naïve neurons and the ability to self-interact and aggregate [25], thus it might be probable that the proper maintenance of parkin activity might protect against the toxic spread of ASN. Since, as parkin often co-localizes with ASN inclusions in PD patient’s brain tissue [3, 26], and many posttranslational modifications of parkin are associated with the toxic conditions evoked by ASN, it is proposed that ASN may affect parkin catalytic activity, solubility, substrate selection, and subcellular localization. Therefore, we aimed at exploring pathological interactions between ASN and parkin, especially the role of extracellular ASN in deregulating parkin levels, posttranslational modifications, and activities.

## Materials and Methods

### Reagents

Dulbecco’s modified Eagle’s medium (DMEM), fetal bovine serum (FBS), horse serum (HS), penicillin, streptomycin, G418, l-glutamine, dimethyl sulfoxide (DMSO), *N*-acetylcysteine (NAC), *N*-ω-nitro-l-arginine (NNLA), polyethylenoimine (PEI), collagen, pluronic, probenecid, Hank’s balanced salt solution (HBSS), Hepes, ethanol, phosphate-buffered saline (PBS), Thioflavin-T, sodium nitroprusside (SNP), D184 (Deano), TRI reagent, deoxyribonuclease I (DNase I), 2′-(4-hydroxyphenyl)-5-(4-methyl-1-piperazinyl)-2,5′-bi-1H-benzimidazole trihydrochloride hydrate, bis Benzimide (Hoechst 33258), 3-(4,5-dimethyl-2-tiazolilo)-2,5-diphenyl-2H-tetrazolium bromide (MTT), Tris-buffered saline, NaCl, Tween 20, 2,2′,2″,2″′-(ethane-1,2-diyldinitrilo)tetraacetic acid (EDTA), sodium fluoride (NaF), sodium orthovanadate (Na_3_VO_4_), sodium pyrophosphate (NaP_2_O_3_), benzamidine, Nonidet P-40, phenylmethylsulfonyl fluoride (PMSF), N-p-Tosyl-l-phenylalanine chloromethyl ketone (TPCK), soybean trypsin inhibitor (STI), aprotinin, leupeptin, and bovine serum albumin (BSA) were obtained from Sigma-Aldrich (St. Louis, MO, USA). Cell Lysis Buffer (10×) was obtained from Cell Signaling (Beverly, MA, USA).

### Preparation of ASN Oligomers and Protofibrils

Human recombinant lyophilized ASN was obtained from rPeptide (Bogart, GA, USA). ASN oligomers were prepared according to Danzer et al. [27] with modifications. Briefly, lyophilized ASN was dissolved to 7 μM concentration in 50 mM sodium phosphate buffer (PB), pH 7.0, containing 20% ethanol. After 4 h of shaking (1000 rpm, room temperature; RT), oligomers were re-lyophilized and re-suspended with one-half of starting volume of 50 mM PB, pH 7.0, containing 10% ethanol. This was followed by stirring, with open lids to evaporate residual ethanol, for 24 h at RT under the sterile hood. Concentration of obtained ASN oligomers was then measured using NanoDrop 2000 spectrophotometer (Thermo Scientific). For preparation of protofibrils, ASN was dissolved in PBS at a concentration of 100 μM and aggregated for 14 days at 37 °C.

### Thioflavin-T Assay

ASN oligomer and aggregate formation was monitored with Thioflavin-T binding fluorescence. Mixture of ASN species with Thioflavin-T (20 μM) was prepared in 50 mM phosphate buffer (50 μl). The sample was kept for 5 min at RT and protected from light. Emission was measured at 483 nm with an excitation at 450 nm using fluorescence spectrophotometer (FLUOstar Omega; BMG LABTECH, Ortenberg, Germany).

### Transmission Electron Microscopy

Sample preparation for transmission electron microscopy (TEM) was performed at RT. Typically, 5 μl of ASN species was applied to the copper mesh covered by formvar and incubated for 1 min. Subsequently, the grid was washed by three drops of deionized water and stained with 2% uranyl acetate for 1 min. The reagent was rinsed with deionized water and dried in the air grid. The analysis was made in the transmission electron microscope Zeiss LEO.

### Atomic Force Microscopy

Sample preparation for atomic force microscopy (AFM) was performed at RT. Typically, 3–6 μl of different ASN species diluted in corresponding buffers to a working concentration of 1 μM were applied to a freshly cleaved muscovite mica substrate (Ted Pella, Redding, CA) and incubated for 1 min. The mica surface was then rinsed with 7–200 μl of double-processed tissue culture water (Sigma-Aldrich, St. Louis, MO, USA) to remove salts and loosely bound proteins. AFM images were recorded on a MultiMode^TM^ SPM (Digital Instruments, Santa Barbara, CA) equipped with an E-Scanner using etched silicon NanoProbes (model RTESP; Veeco Instruments, Mannheim, Germany). All measurements were performed in the tapping mode with scan rates of ~ 0.5 Hz. Images were processed using NanoScope software (Digital Instruments).

### Cell Culture

The studies were carried out using rat pheochromocytoma (PC12) cells. PC12 cells were cultured in DMEM supplemented with 10% heat-inactivated FBS, 5% heat-inactivated HS, 50 units/ml penicillin, and 50 μg/ml streptomycin and l-glutamine at 37 °C in a humidified incubator in 5% CO_2_ atmosphere.

### Cellular Treatment

PC12 cells were seeded into 60- or 35-mm culture dishes, 24-well or 96-well plates (coated with 0.1% PEI or rat tail collagen), and the growth medium was changed into a low-serum medium (DMEM supplemented with 2% FBS, 1% penicillin/streptomycin, and 1% l-glutamine). HBSS or other media appropriate for the particular procedure were also used. Then, the cells were treated with exogenous ASN oligomers (5 μM), NAC (100 μM; dissolved in water), and NNLA (100 μM; dissolved in water) for appropriate time points. Suitable solvent was added to respective controls.

### siRNA-Mediated Parkin Knock-Down

For RNA interference, PC12 cells were transfected with appropriate siRNA: PARK-2 (L-090709-02; Dharmacon) or control (D-001810-10-05; Dharmacon) using Lipofectamine RNAiMAX (Invitrogen) according to the manufacturer’s protocol. The expression of parkin in transfected cells was then examined by RT-PCR and Western blot.

### Stable, Constitutive Overexpression of Parkin in PC12 Cells

The sequence encoding human wild-type parkin was subcloned into AscI/PacI sites of pcDNA4.3Asc vector. The construct was evaluated first using AscI/PacI restriction analysis and then with Western blot on extracts of transiently transfected CHO cell with antibodies against parkin (Santa Cruz, SC-32282).

PC12 cells were electroporated at 5 × 10^6^ per cuvette (Bio-Rad Gene Pulser Xcell) with 10 μg of DNA (0.5 ml medium), using VWR cuvettes with 4 mm gap and one 30 ms pulse of 220 V. The cells were plated in culture medium and propagated with one medium change until reaching 90% confluency. Then, selection was started using G418 (30 μg/ml initial concentration, gradually increased to 100 μg/ml). After the cells accumulated, clonal selection was performed.

### Fluorometric Measurements of Changes in [Ca^2+^]_i_

Changes in intracellular Ca^2+^ ([Ca^2+^]_i_) concentration in PC12 cells were monitored using the fluorescent calcium-sensitive probe Fluo-4. Its acetoxymethyl ester derivative, Fluo-4AM, easily penetrates plasma membranes, and inside the cells it is cleaved by esterases to Fluo-4, which becomes highly fluorescent after binding with Ca^2+^. The procedure is essentialy as described previously by Wilkaniec et al. [28]. PC12 cells were seeded onto collagen-coated 96-well dark plates at the density of 1.4 × 10^5^ cells/ml. After 24 h, the cells were loaded with 10 μM Fluo-4AM supplemented with 0.02% Pluronic® F-68 for 60 min at 37 °C in a standard HBSS. The cells were washed three times with HBSS and, to ensure complete AM ester hydrolysis, kept for 30 min at 37 °C in the dark. After a second washing, the fluorescence was measured using a microplate reader FLUOstar Omega (Ortenberg, Germany) set at 485 nm excitation and 538 nm emission wavelengths. After determining the baseline fluorescence of the cells incubated in HBSS, the changes in fluorescence after the addition of the test compounds were recorded every 15 s for 5 min. This 5-min treatment did not have any significant impact on cell viability. The results of fluorescence measurements are presented as percent changes in fluorescence intensity relative to the basal level versus duration of measurement (%*F*/*F*0). To quantify the change in the dynamics of the Ca^2+^ responses, the area under the curve (AUC) was calculated as a measure for the increase in intracellular Ca^2+^.

### HPLC Measurements of Arginine and Its Metabolites

Cells were scraped in 1 ml of PBS, spun down, and re-suspended in 100 μl of 45 mM phosphate buffer pH 6.2 with 10% acidic methanol. After sonication (10 cycles 3 s each, 10% power setting, BioLogics 150V/T), the material was centrifuged for 10 min at 13,000×*g*. The protein pellet was re-suspended in 0.5 ml 1 M NaOH for protein measurement (Bradford). Supernatant was stored at − 20 °C and used for HPLC amino acid analysis with fluorescence detection after derivatization in a timed reaction with *o*-phtalaldehyde plus mercaptoethanol, as described by Jesko et al. [29]. Derivatized samples (25 μl) were injected on to a 150 × 4.6-mm 5 μ Hypersil Gold BDS C18 column with a mobile phase of 50 mM phosphate buffer (KH_2_PO_4_/K_2_HPO_4_) containing 10% *v*/*v* methanol, pH 6.2 (solvent A), and methanol (solvent B).

### Measurement of Intracellular Free Radical Level

Measurement of the free radicals level was carried out using fluorescent indicator 2′7’-dichlorofluorescein diacetate (DCFH-DA) (Cayman Chemical Company), as described previously [6]. DCFH-DA is intracellularly deacetylated to 2′7′-dichlorofluorescin (DCFH) and then oxidized by hydrogen peroxide to a fluorescent compound, 2′7’-dichlorofluorescein (DCF). PC12 cells were incubated in DCFH-DA (10 μM) solution in HBSS with 20 mM Hepes (pH 7.4) and 0.02% Pluronic for 50 min at 37 °C in the dark. Then, the cells were washed three times and the DCF fluorescence was measured using a microplate reader FLUOstar Omega (Ortenberg, Germany) at 485 nm excitation and 538 nm emission wavelengths. After determining the baseline fluorescence of the cells incubated in HBSS, the changes in fluorescence after the addition of the test compounds were recorded every 1 for 8 h. The results of fluorescence measurements are presented as percent of corresponding control.

### Determination of Intracellular Nitric Oxide Level in Cells

Measurement of the nitric oxide level was carried out using fluorescent indicator 4,5-diaminofluorescein diacetate (DAF-2 DA) (Cayman Chemical Company). DAF-2 DA is oxidized by nitric oxide to a fluorescent compound, DAF-2. PC12 cells were incubated 20 min at 37 °C in the dark with 10 μM DAF-2 DA in the presence of 0.02% Pluronic. The cells were washed with Pluronic-supplemented Hanks’ balanced salt solution with 20 mM Hepes (pH 7.4) and kept for 30 min at 37 °C in the dark. After a second washing, the fluorescence was measured using a microplate reader FLUOstar Omega (Ortenberg, Germany) set at 488 nm excitation and 530 nm emission wavelengths. After determining the baseline fluorescence of the cells incubated in HBSS, the changes in fluorescence after the addition of the test compounds were recorded every 1 for 8 h. The results of fluorescence measurements are presented as percent of corresponding control.

### Determination of Nitric Oxide Level in Cell-Free System

Measurement of the nitric oxide level was carried out using fluorescent indicator DAF-2 DA (Cayman Chemical Company). Mixture of methanol, 2.5 mM DAF-2 DA, and 2 M KOH (1:1:0.5) was kept in darkness at RT for 1 h. Then, HCl was added to neutralize the mixture (pH 7.0). NAC (100 μM), NNLA (100 μM), SNP (500 μM), Deano (500 μM), water, and DAF-2 DA (10 μM) were added to selected well. The fluorescence of DAF-2 was measured by 8 h using a fluorescence spectrophotometer (FLUOstar Omega; BMG LABTECH, Ortenberg, Germany) with excitation at 488 nm and emission at 530 nm.

### Cytosolic Redox Environment

To investigate changes in cytosolic redox environment, PC12 cells were transfected with a plasmid coding for a redox-sensitive green fluorescent protein (roGFP in pEGFP-N1). In an oxidized environment, the absorption increases at short wavelengths (375 nm) at the expense of absorption at longer wavelengths (500 nm). The fluorescence ratio indicates oxidation/reduction as described previously by Cannon and Remington [30]. PC12 cells were transfected using electroporation (Neon Transfection System) in 100 μl volume containing 1.4 × 10^6^ cells and 20 μg DNA, at manufacturer’s PC12-optimized pulse parameters (ThermoFisher Scientific). Cells were plated in four replicates onto 96-well plates at a density of 1.5 × 10^4^ cells/well in standard culture medium less antibiotics and kept overnight at 37 °C in 5% CO_2_. After 24-h treatment with oligomeric ASN, cells were washed twice with PBS and placed in a Hank’s buffer. The ratio 375 nm/500 nm was measured using multiplate reader Infinite M1000 PRO (TECAN). An increase of the ratio indicates a more oxidized environment.

### Quantitative Real-Time Polymerase Chain Reaction

The total RNA isolation was performed according to the procedure developed by Chomczyński, using TRI Reagent® (cat. T9424) from Sigma-Aldrich, following the manufacturer’s protocol. Digestion of DNA contamination was performed using DNase I according to the manufacturer’s protocol (Sigma-Aldrich, St. Louis, MO, USA). RNA quantity and quality were controlled by spectrophotometric analysis and gel electrophoresis. A reverse transcription was performed by using the high capacity cDNA reverse transcription kit according to the manufacturer’s protocol (Applied Biosystems, Foster City, CA, USA). Quantitative real-time PCR was performed with TaqMan Universal PCR Master Mix (Applied Biosystems, Foster City, CA, USA) or Power SYBR Green PCR Master Mix (Applied Biosystems, Foster City, CA, USA) and detected by a Real-Time PCR System on an ABI PRISM 7500 apparatus (Thermo Fisher Scientific, Waltham, MA, USA) using the commercially available TaqMan® Gene Expression Assays (*Actb* Rn01412977_g1; *Park-2* Rn00571787_m1; *Gpx1*Rn00577994_g1, *Txnrd1* Rn01503798_m1) or selected primer pairs (forward/reverse): *Sod2* 5′-CGCTGGCCAAGGGAGAT-3′/5′-CCCCGCCATTGAACTTCA-3′; *Gadd45*5′-CGGGACCGGGACATCTC-3′/5′-GGCACTTCAGGGCTTTCTCTT-3′; *Prdx3* 5′-GTGGATTCCCACTTCAGTCATCT-3′/5′-GTTCATGTGGCCCAAACCA-3′; and *Top1mt* 5′-CGACTGGCAGAAGGAAATGAC-3′/5′-AGTGCCTATGGATCTCCGAGAA-3′ for SYBR Green assay. Actb was used in the analysis as a reference gene. A standard two-step PCR amplification was performed, with a melting step at 95 °C for 15 s and annealing and elongation at 60 °C for 1 min, for 40 cycles. For SYBR Green assay, after PCR amplification, a first derivative melting curve analysis was conducted to confirm the specificity of the PCR. The relative levels of target mRNA, normalized to an endogenous reference and relative to a calibrator, were calculated by 2^–ΔΔCT^ formula.

### Parkin Co-Immunoprecipitation

The cells were washed three times with ice-cold PBS and lysed in cold lysis buffer containing 50 mM Tris-HCl (pH 7.4), 0.25 M NaCl, 0.1% *v*/*v* Nonidet P-40, 5 mM EDTA, 50 mM NaF, 1 mM Na_3_VO_4_, 1 mM Na_4_P_2_O_7_, 10 mM benzamidine, 50 μg/ml PMSF, 10 μg/ml TPCK, 10 μg/ml STI, 1 μg/ml aprotinin, and 1 μg/ml leupeptin. Protein levels were determined using the Bradford method, and 1 mg of protein lysate was combined with 25 μl Protein G-Dynabeads (Novex, Life Technologies) pre-incubated with mouse anti-parkin (1 μg) antibody followed by overnight incubation by rotation at 4 °C. Cell lysates were rotated with antibody-bound Dynabeads at 4 °C for 1 h, and the obtained complexes were sequentially washed three times with IP buffer. Immunoprecipitates were eluted by heating at 95 °C for 5 min in 2× Laemmli sample buffer and separated on SDS/PAGE gels, transferred to nitrocellulose membranes at 100 V, and subjected to Western blot analysis.

### Western Blot Analysis

The cells were washed twice with ice-cold PBS and lyzed in Cell Lysis Buffer (1×). Protein levels were determined using the Bradford method, and then the samples were mixed with Laemmli buffer and denatured at 95 °C for 5 min. Equal amounts of proteins were separated on Native-PAGE gels (ASN preparations) or SDS/PAGE gels (cell lysates or immunoprecipitates). All proteins were transferred to nitrocellulose membranes at 100 V. Membranes were washed for 5 min in TBS-Tween buffer (0.1% TBST) (100 mM Tris-buffered saline, 140 mM NaCl, and 0.1% Tween 20, pH 7.6) and the non-specific bindings were blocked for 1 h at RT with 5% BSA in 0.1% TBST or with 5% non-fat milk solution in 0.1% TBST. Immunodetection was performed overnight at 4 °C using mouse anti-parkin (1:1000; Santa Cruz), rabbit anti-parkin (1:500; Cell Signaling), rabbit anti-alpha-synuclein (1:500; Sigma-Aldrich), rabbit anti-*S*-nitrosocysteine (1:1000; Sigma-Aldrich), or rabbit anti-ubiquitin (1:1000; Millipore) antibodies. Then, the membranes were washed three times (5 min) in TBST and incubated for 60 min at RT with secondary antibody (anti-rabbit or anti-mouse IgG) (1∶4000) in a 5% non-fat milk/TBST. Antibodies were detected using chemiluminescent Clarity Western ECL Substrate (Bio-Rad Laboratories, Hercules, CA, USA) under standard conditions. Immunolabeling of GAPDH (rabbit anti-GAPDH; 1:40,000; Sigma-Aldrich) for cell lysates or immunolabeling of rabbit anti-parkin for immunoprecipitates was performed as a loading control.

### Parkin In Vitro Autoubiquitination Assay

Autoubiquitination of parkin was analyzed according to Yao et al. [10] with modifications. Briefly, parkin was immunoprecipitated with murine anti-parkin antibody, as described above. Immunoprecipitate was incubated in the presence of ubiquitin (0.02 mg/ml), ubiquitin-activating enzyme Ube1 (5 nM), and ubiquitin-conjugating enzyme UbcH4 (100 nM) in ubiquitination buffer (2 mM ATP, 50 mM Tris, 5 mM MgCl_2_ at pH 8) for 30 min at 37 °C. Reaction was stopped by addition of 50 μl of 2× concentrated Laemmli sample buffer and denaturation was performed for 5 min at 95 °C. After separation of beads on magnetic stand, samples were analyzed by SDS-PAGE and Western blotting with rabbit anti-ubiquitin antibody and rabbit anti-parkin antibody. Anti-ubiquitin immunoreactivity was normalized to anti-parkin immunoreactivity.

### Cell Viability and Apoptosis

Cellular viability was evaluated by the reduction of MTT to formazan. Low-serum medium containing investigated substances were added to the cells for 48 h. MTT (2.5 mg/ml) was added to all wells and allowed to incubate at 37 °C for 2 h, followed by cell lysis and spectrophotometric measurement at 595 nm.

The presence of apoptotic cells was determined by microscopic analysis of the cells stained with 2′-(4-hydroxyphenyl)-5-(4-methyl-1-piperazinyl)-2,5′-bi-1H-benzimidazole trihydrochloride hydrate, bisBenzimide (Hoechst 33258). Cells with typical apoptotic nuclear morphology (nuclear shrinkage, condensation) were identified and counted. The results were expressed as percentages of apoptotic bodies.

### Statistical Analysis

The results were expressed as mean values ± SEM. Differences between the means were analyzed using a Student’s *t* test between two groups and one-way or two-way analysis of variance ANOVA with Bonferroni comparison post hoc test among multiple groups. Statistical significance was accepted at *p* < 0.05. The statistical analyses were performed using GraphPad Prism version 5.0 (GraphPad Software, San Diego, CA).

## Results

**Fig. 1:**
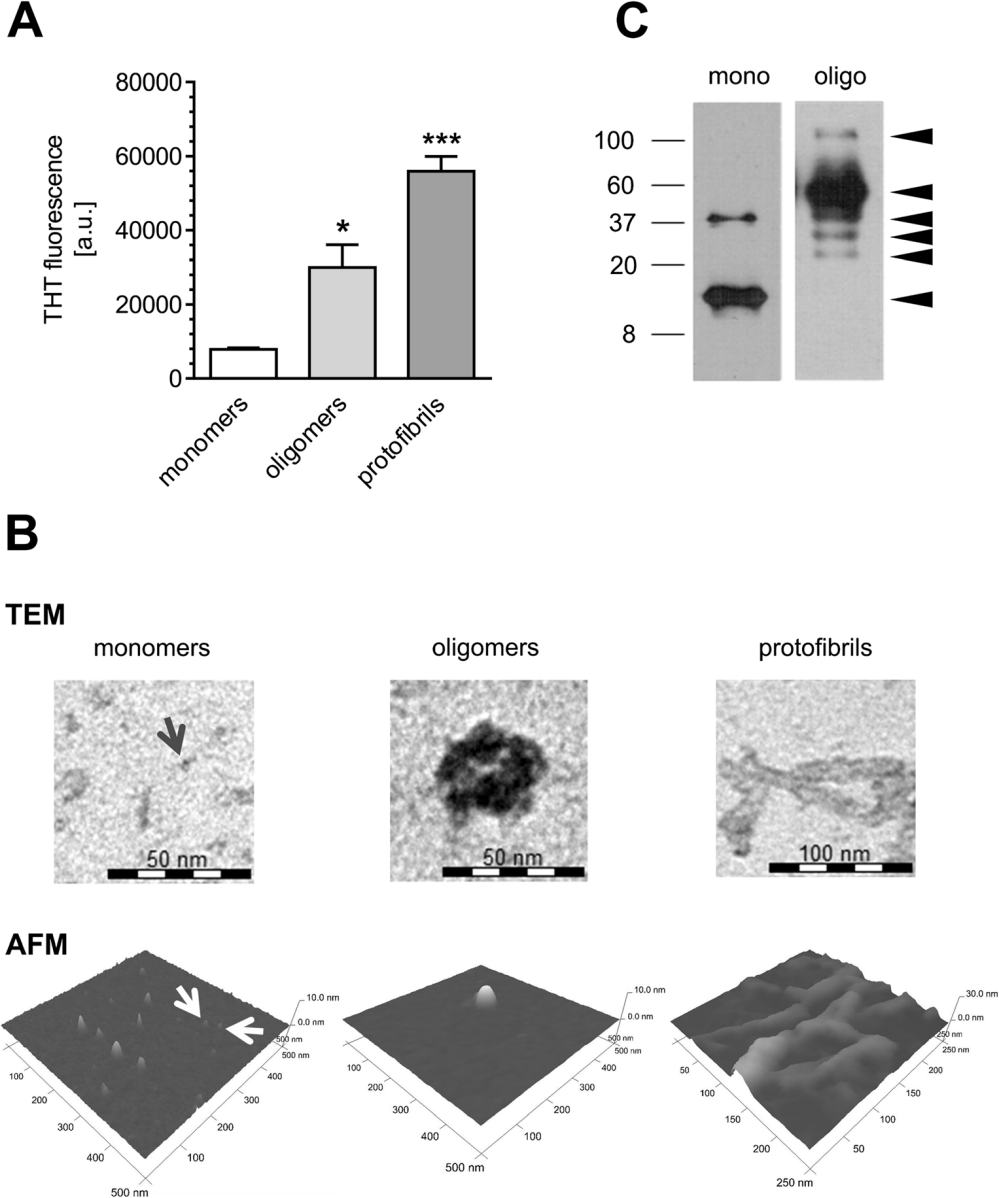
Characterization of ASN oligomer structure. **a** The oligomerization and fibrillization of ASN species (monomers, oligomers, and protofibrils) measured by Thioflavin-T (ThT) fluorescence. Data represent the mean value ± SEM for three independent experiments. *p < 0.05; ***p < 0.001 compared to ASN monomers, using one-way ANOVA followed by Bonferroni post hoc test. **b** The oligomerization and fibrillization of ASN species (monomers, oligomers, and protofibrils) visualized by transmission electron microscopy (TEM) and atomic force microscopy (AFM). AFM and TEM images are representative examples of several images of independent ASN species preparations. Red arrows on TEM and AFM pictures show single ASN monomers. **c** Native gel electrophoresis and blotting with anti-ASN antibody of the ASN monomeric (mono) and oligomeric (oligo) forms were performed. The molecular masses of the particular bands (delineated by arrowheads) corresponding to monomeric and oligomeric states of ASN were computed using Total Lab software. Non-aggregated ASN mostly remains in the monomeric form (molecular mass 11 kDa), but to a small extent, it forms dimers and trimers with a molecular mass of 37 kDa. In the oligomeric preparations of ASN, no immunoreactive bands corresponding to monomeric forms was detected; however, the bands with apparent higher molecular masses of 25, 33, and 37 (characteristic for dimers and trimers), as well as 51 and 106 kDa (larger oligomers) were resolved

Since oligomers and protofibrils, rather than mature fibrils of ASN, were previously shown to be the most pathogenic species involved in neurodegeneration [25, 31, 32], we generated ASN oligomeric species and confirmed their conformation state by measuring Thioflavin-T (ThT) fluorescence response upon binding to appropriate variants. We found that the ThT fluorescence is four and seven times more intense with ASN oligomers and protofibrils, respectively, compared to monomers (Fig. 1a). For morphological characterization of obtained preparations, TEM and AFM analyses were performed according to Danzer et al. [27]. We observed that ASN oligomers formed annular structures 40–45 nm in diameter. Importantly, these annular structures were not found in monomer preparations. ASN monomers showed an amorphous ultrastructure and, and as expected, ASN fibrils showed a fibrillar helical ultrastructure (Fig. 1b). Native-page electrophoresis and Western blot analysis revealed that ASN oligomers had assembled into supramolecular structures, much larger than monomers (Fig. 1c).

We first investigated whether those in vitro generated oligomeric species are able to activate the molecular mechanisms responsible for neuronal cells death. As an experimental model, we chose rat pheochromocytoma (PC12) cell line, because these cells are able to express a number of features characteristic for catecholaminergic neurons including tyrosine hydroxylase and dopamine-β-hydroxylase activities and were used in previous studies on mechanisms of ASN-induced cytotoxicity [6, 33, 34].

**Fig. 2:**
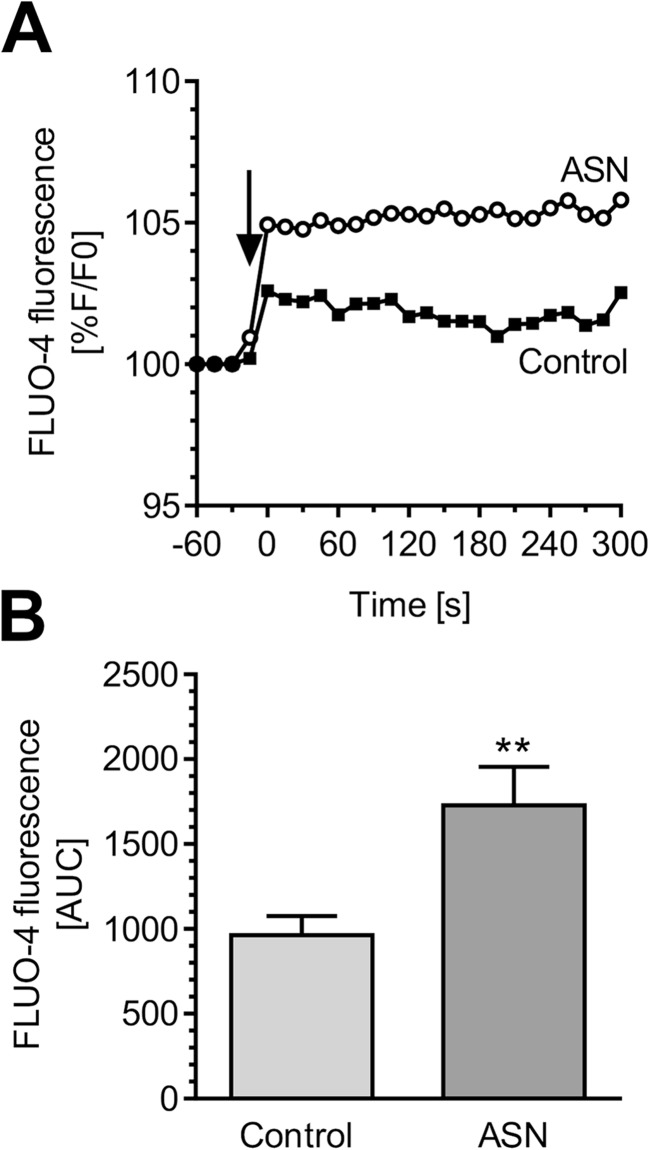
The effect of ASN oligomers on calcium homeostasis in PC12 cells. **a** Cytoplasmatic calcium level in PC12 cells after 6 min treatment with 5 μM ASN oligomers measured by FLUO-4 fluorescence. Arrow indicates compound administration. Data represent the mean value ± SEM for three independent experiments. **b** Responses of FLUO-4 were quantitated by measuring the area under the curve (AUC) value. Data represent the mean value ± SEM for three independent experiments. **p < 0.01 compared to control using Student’s *t* test

**Fig. 3:**
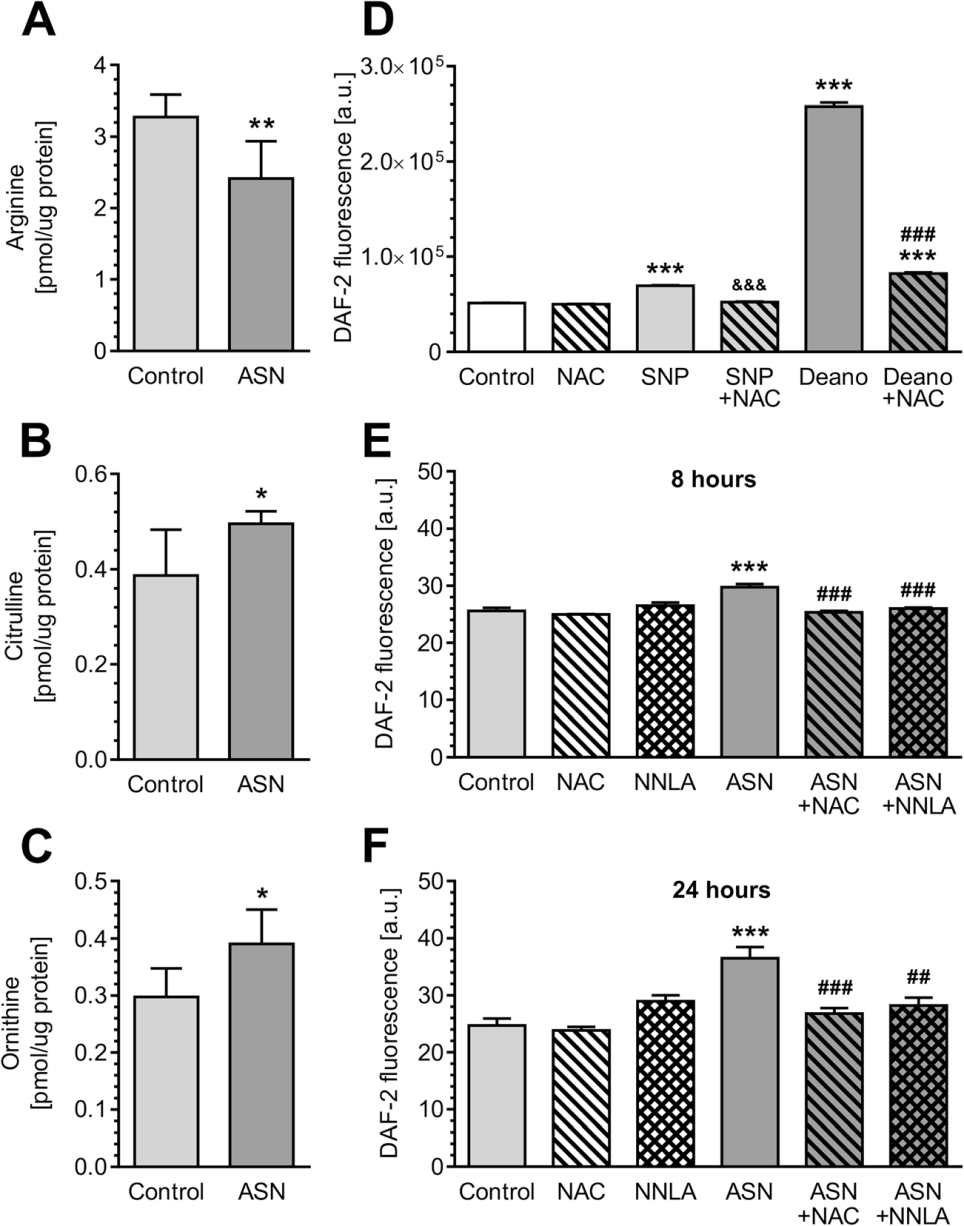
ASN oligomers increase nitric oxide generation in PC12 cells. **a** Arginine levels in PC12 cells after 24-h incubation with 5 μM ASN oligomers, as measured by HPLC method. Data represent the mean value ± SEM of five independent experiments. **p < 0.01 compared to control, using Student’s *t* test. **b** Citrulline level in PC12 cells after 24-h incubation with 5 μM ASN oligomers measured by HPLC method. Data represent the mean value ± SEM of five independent experiments. *p < 0.05 compared to control, Student’s *t* test. **c** Ornithine levels in PC12 cells after 24-h incubation with 5 μM ASN oligomers measured by HPLC method. Data represent the mean value ± SEM of five independent experiments. *p < 0.05 compared to control, Student’s *t* test. **d** NO levels after 2-h incubation with 1 mM *N*-acetylcysteine (NAC), 500 μM sodium nitroprusside (SNP), and 500 μM Deano measured by DAF-2 fluorescence. Data represent the mean value ± SEM for four independent experiments. ***p < 0.001 compared to control, ^&&&^p < 0.001 compared to SNP, ###*p* < 0.001 compared to Deano, using one-way ANOVA followed by Bonferroni post hoc test. **e** Intracellular NO level in PC12 cells after 8-h incubation with 5 μM ASN oligomers in the presence of 1 mM NAC or 100 μM NNLA measured by DAF-2 fluorescence. Data represent the mean value ± SEM for six independent experiments. ***p < 0.001 compared to control; ###*p* < 0.001 compared to ASN, using one-way ANOVA followed by Bonferroni post hoc test. **f** Intracellular NO level in PC12 cells after 24-h incubation with 5 μM ASN oligomers in the presence of 1 mM NAC or 100 μM NNLA measured by DAF-2 fluorescence. Data represent the mean value ± SEM for six independent experiments. ***p < 0.001 compared to control; ###*p* < 0.001; ##*p* < 0.01 compared to ASN, using one-way ANOVA followed by Bonferroni post-hoc test

In the present study, we exposed PC12 cells to generated oligomers to study their influence on cellular calcium homeostasis and oxidative stress. We observed that treatment of PC12 cells with ASN oligomers (5 μM) resulted in significant [Ca^2+^]_i_ mobilization (Fig. 2a, b). Since calcium deregulation plays an important role in overactivation of nitric oxide synthase (NOS) that catalyzes the conversion of arginine into citrulline and NO, we next examined the level of the corresponding amino acids as well as intracellular NO levels. Our results showed that arginine was significantly decreased in PC12 cells treated with extracellular ASN together with the moderate increase in citrulline levels (Fig. 3a, b). Since arginine levels are also regulated by the activity of arginase, we also measured the level of the arginase product ornithine and observed that this was also significantly increased after ASN treatment (Fig. 3c). In the following experiments, we measured NO levels using the fluorescence dye DAF-2 DA. To determine the contribution of ASN oligomers on nitrosative stress, we used NAC, an antioxidant that also holds a high NO scavenging potency. As shown in cell-free system, NAC (100 μM) decreased DAF-2 fluorescence intensity induced by NO donors, SNP (500 μM) or Deano (500 μM) (Fig. 3d). In PC12 cells, we found that ASN oligomers evoked a significant elevation in NO synthesis after 8 h of incubation (Fig. 3e). The progressive rise of the NO level in ASN-treated cells was observed even up to 24 h (Fig. 3f). Subsequently, pre-treatment of PC12 cells with NAC (100 μM) or unselective NOS inhibitor, NNLA (100 μM), significantly prevented the increase of NO level induced by ASN (Fig. 3e, f).

**Fig. 4:**
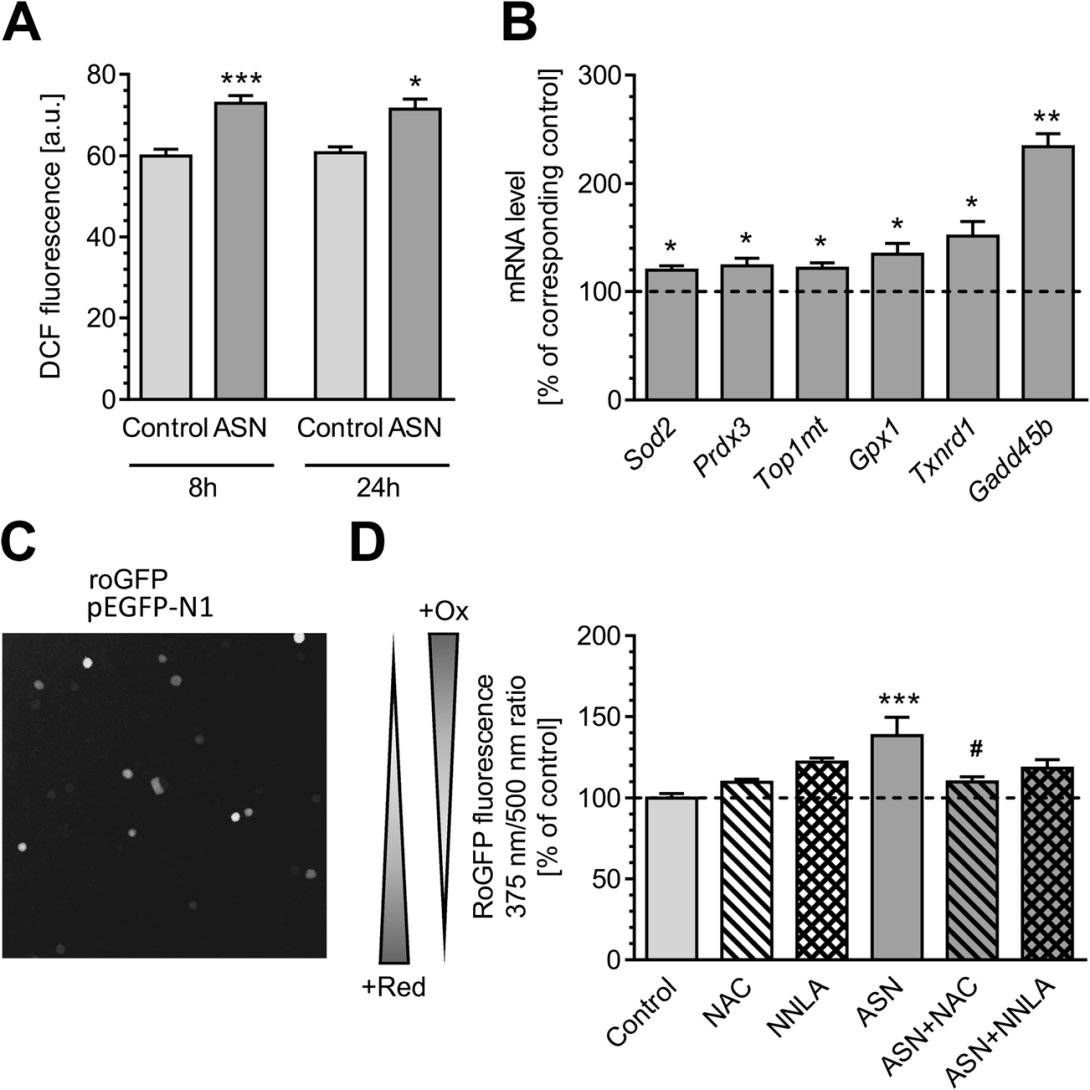
The effect of ASN oligomers on oxidative stress generation in PC12 cells. **a** Intracellular free radicals level in PC12 cells after 8- and 24-h incubation with 5 μM ASN oligomers measured by DCF fluorescence. Data represent the mean value ± SEM for four independent experiments. ***p < 0.001; *p < 0.05 compared to control, using Student’s *t* test. **b** Expression of stress response genes: *Sod2*, *Prdx3*, *Top1mt*, *Gpx1*, *Txnrd1*, and *Gadd45b* in PC12 cells after 24-h treatment with 5 μM ASN oligomers measured by qRT-PCR. Data represent the mean value ± SEM for four independent experiments. **p < 0.01; *p < 0.05 compared to control, using a Student’s *t* test. **c** Representative confocal microscope image of PC12 cells transiently overexpressing roGFP protein. **d** Oxidative-reduction potential in PC12 cells after 8-h incubation with 5 μM ASN oligomers in the presence of 1 mM NAC or 100 μM NNLA measured by RoGFP fluorescence. Data represent the mean value ± SEM for five independent experiments. ***p < 0.001 compared to control, #*p* < 0.05 compared to ASN, using one-way ANOVA followed by Bonferroni post hoc test

Our previous findings indicated that ASN-induced mitochondrial dysfunction was responsible for elevated oxidative stress [6]. In agreement with these earlier findings, cytosolic ROS level assayed by the DCF method was significantly elevated in PC12 cells treated with extracellular ASN for 8 and 24 h as compared to control PC12 cells (Fig. 4a). Simultaneously, we observed that ASN oligomers increase the expression of genes encoding stress-activated antioxidative enzymes localized in mitochondria: superoxide dismutase (*Sod2*), peroxiredoxin 3 (*Prdx3*), topoizomerase 1 (*Top1mt*); or in cytosol: glutathione peroxidase 1 (*Gpx1*) and thioredoxin reductase 1 (*Txnrd1*). An increased expression of Gadd45β as a marker of cellular stress and DNA-damage was also observed in ASN-treated PC12 cells (Fig. 4b). To verify that the cytosolic redox environment was affected by the increase in ROS, PC12 cells were transiently transfected with a reporter gene coding for a roGFP (Fig. 4c) and treated with ASN oligomers for 24 h. The results indicated that ASN significantly deregulates cellular redox state in PC12 cells. Moreover, this effect by ASN was significantly ameliorated by NAC pre-treatment (Fig. 4d).

**Fig. 5:**
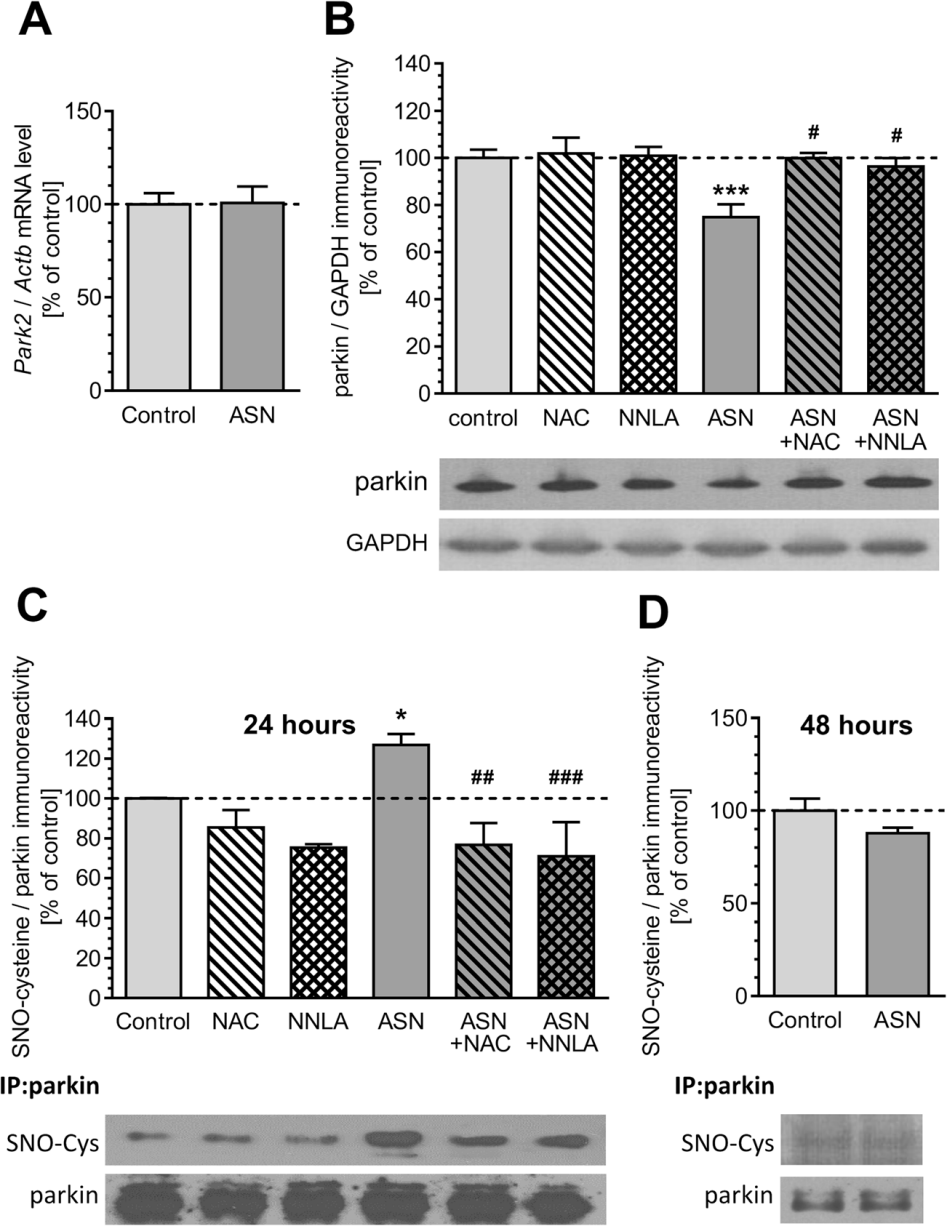
The effect of ASN oligomers on parkin expression and *S*-nitrosylation in PC12 cells. **a** Expression of *Park2* gene in PC12 cells after 24-h treatment with 5 μM ASN oligomers measured by qRT-PCR. Results were normalized to actin (*ActB*) level. Data represent the mean value ± SEM for four independent experiments (*p* = 0.951 by Student’s *t* test). **b** Parkin immunoreactivity in PC12 cells after 24-h treatment with 5 μM ASN oligomers in the presence of 1 mM NAC or 100 μM NNLA measured by Western blot. Results were normalized to GAPDH level. Data represent the mean value ± SEM for four independent experiments. ***p < 0.001 compared to control; #*p* < 0.05 compared to ASN, using one-way ANOVA followed by Bonferroni post hoc test. **c** Parkin *S*-nitrosylation in PC12 cells after 24-h treatment with 5 μM ASN oligomers in the presence of 1 mM NAC or 100 μM NNLA determined by ratio of *S*-nitrosocysteine (SNO-cysteine)/parkin immunoreactivity, measured by Western blot. Results were normalized to parkin level. Data represent the mean value ± SEM for five independent experiments. *p < 0.05 compared to control, ##*p* < 0.01; ###*p* < 0.001 compared to ASN, using one-way ANOVA followed by Bonferroni post hoc test. **d** Parkin *S*-nitrosylation in PC12 cells after 48-h treatment with 5 μM ASN oligomers determined by the ratio of *S*-nitrosocysteine (SNO-cysteine)/parkin immunoreactivity, measured by Western blot. Results were normalized to parkin level. Data represent the mean value ± SEM for five independent experiments (*p* = 0.107 by Student’s *t* test)

Because nitrosative and oxidative stress may affect parkin function, we investigated the effect of ASN treatment on parkin expression, *S*-nitrosylation, as well as its activity. Although ASN did not alter parkin mRNA levels (Fig. 5a), it significantly lowered parkin immunoreactivity (Fig. 5b). This effect of ASN on parkin protein levels was prevented by both NAC and NNLA (Fig. 5b). Western blot analysis of cysteine nitrosylation in immunoprecipitated parkin revealed that *S*-nitrosylation of parkin in PC12 cells increases within 24 h of ASN oligomer exposure (Fig. 5c), whereas 48 h after ASN treatment, the level of *S*-nitrosylated parkin returned to basal levels (Fig. 5d). Similar to findings in previous experiments, 24-h treatment with NAC and NNLA prevented ASN-evoked parkin *S*-nitrosylation (Fig. 5c).

**Fig. 6:**
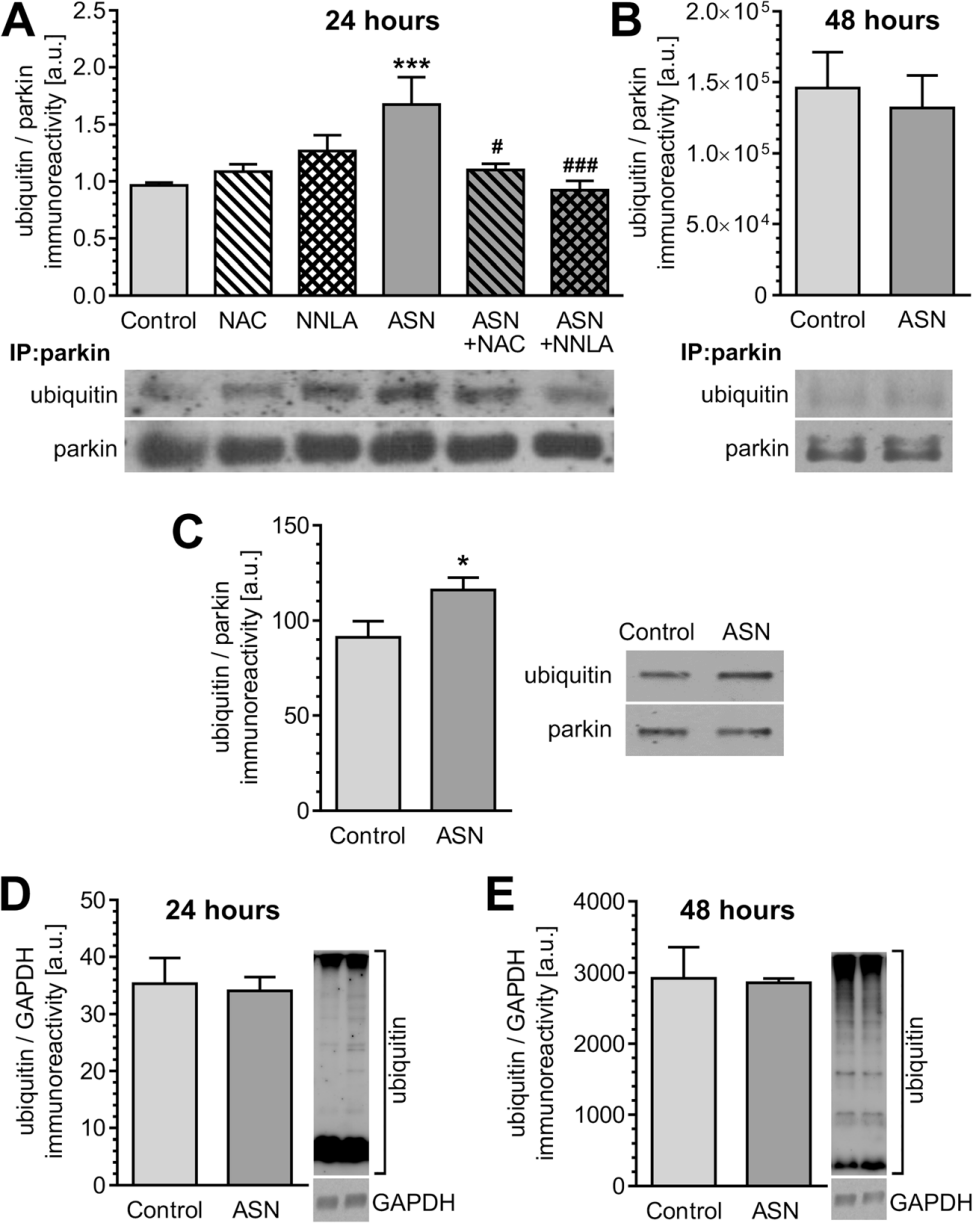
The effect of ASN oligomers on protein ubiquitination and parkin activity in PC12 cells. **a** Parkin ubiquitination in PC12 cells after 24-h treatment with 5 μM ASN oligomers in the presence of 1 mM NAC or 100 μM NNLA measured by Western blot. Results were normalized to parkin level. Data represent the mean value ± SEM for five independent experiments. ***p < 0.001 compared to control, ###*p* < 0.001; #*p* < 0.05 compared to ASN, using one-way ANOVA followed by Bonferroni post hoc test. **b** Parkin ubiquitination in PC12 cells after 48-h treatment with 5 μM ASN measured by Western blot. Results were normalized to parkin level. Data represent the mean value ± SEM for four independent experiments (*p* = 0.703 by Student’s *t* test). **c** Parkin activity in PC12 cells after 24-h treatment with 5 μM ASN determined by the ratio of ubiquitin/parkin immunoreactivity measured by Western blot. Results were normalized to parkin level. Data represent the mean value ± SEM for five independent experiments. *p < 0.05 compared to control, using Student’s *t* test. **d** Protein ubiquitination in PC12 cells after 24-h treatment with 5 μM ASN measured by Western blot. Results were normalized to GAPDH level. Data represent the mean value ± SEM for five independent experiments (*p* = 0.811 by Student’s *t* test). **e** Protein ubiquitination in PC12 cells after 48-h treatment with 5 μM ASN measured by Western blot. Results were normalized to GAPDH level. Data represent the mean value ± SEM of four independent experiments (*p* = 0.894 by Student’s *t* test)

We next determined whether ASN treatment modulated the ubiquitin E3 ligase activity of parkin. Parkin ubiquitinates itself; therefore, detection of ubiquitinated parkin serves as a reliable indicator of parkin’s ubiquitin E3 ligase activity [35–37]. We found that autoubiquitination of parkin significantly increases after 24-h treatment with ASN oligomers (Fig. 6a) and returns to the basal level at 48 h (Fig. 6b). Pre-treatment with NAC or NNLA significantly prevented ASN-induced elevation in parkin autoubiquitination (Fig. 6a). To further confirm the effect of ASN on parkin’s E3 ligase activity, we reconstituted the autoubiquitination reaction of parkin in vitro. Again, we observed that ASN markedly elevated the activity of parkin (Fig. 6c). Since parkin has many putative substrates, we monitored whether the ASN-induced changes in parkin activity affected the parkin-mediated protein ubiquitination. Although exogenous ASN-induced significant increases in parkin activity, we did not observe any substantial changes in the level of ubiquitinated proteins in PC12 cells treated with exogenous ASN for 24 h (Fig. 6d) or 48 h (Fig. 6e).

**Fig. 7:**
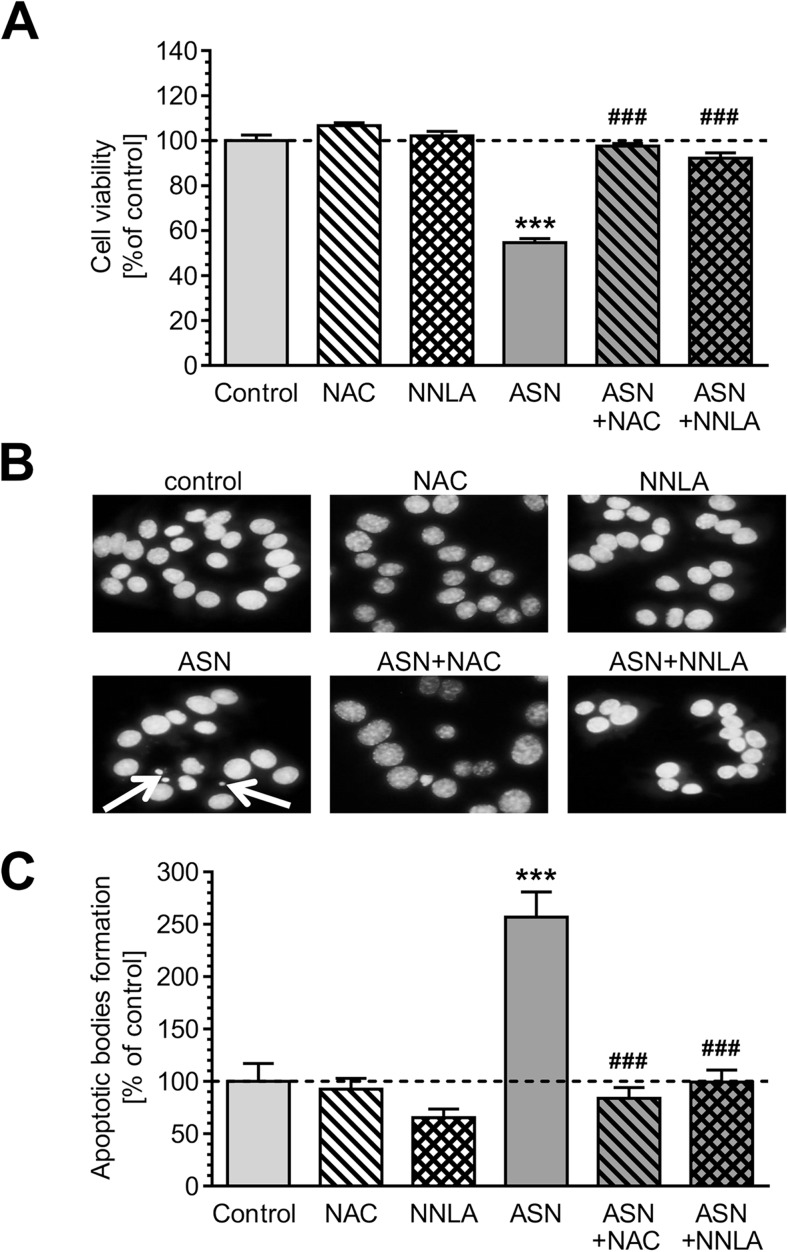
The effect of ASN oligomers on PC12 cells viability. **a** PC12 cell viability after 48-h treatment with 5 μM ASN oligomers in the presence of 1 mM NAC or 100 μM NNLA measured by MTT test. Data represent the mean value ± SEM for five independent experiments. ***p < 0.001 compared to control; ###*p* < 0.001 compared to ASN, using one-way ANOVA followed by Bonferroni post hoc test. **b** Representative photomicrographs of apoptotic body formation in PC12 cells after 48-h treatment with 5 μM ASN oligomers in the presence of 1 mM NAC or 100 μM NNLA measured by Hoechst 33342 staining. The arrows indicate nuclei with typical apoptotic features. **c** Apoptotic body formation in PC12 cells after 48-h treatment with 5 μM ASN oligomers in the presence of 1 mM NAC or 100 μM NNLA measured by Hoechst 33342 staining. Data represent the mean value ± SEM for four independent experiments. ***p < 0.001 compared to control; ###*p* < 0.001 compared to ASN, using one-way ANOVA followed by Bonferroni post hoc test

**Fig. 8:**
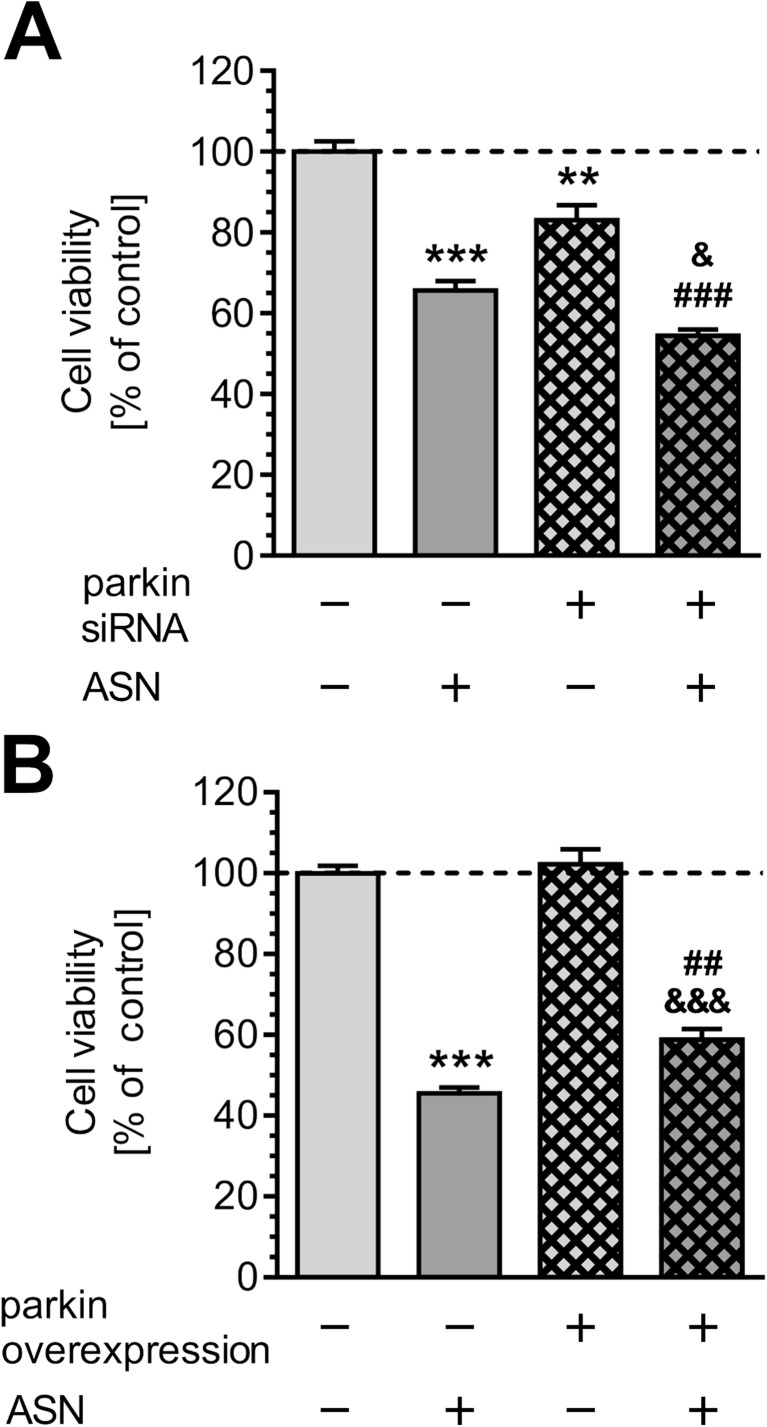
The effect of ASN oligomers on parkin knock-down and parkin overexpression PC12 cell viability. **a** Parkin knock-down PC12 cells viability after 48-h treatment with 5 μM ASN oligomers measured by MTT test. Data represent the mean value ± SEM for five independent experiments. **p < 0.01; ***p < 0.001 compared to control siRNA; ###*p* < 0.001 compared parkin siRNA; ^&^*p* < 0.001 compared to control siRNA + ASN, using two-way ANOVA followed by Bonferroni post hoc test. **b** Parkin overexpressed PC12 cell viability after 48-h treatment with 5 μM ASN oligomers measured by MTT test. Data represent the mean value ± SEM for five separate experiments. ***p < 0.001 compared to control; ^&&&^*p* < 0.001 compared to parkin overexpressed cells; ##*p* < 0.01 compared to control + ASN, using two-way ANOVA followed by Bonferroni post hoc test

ASN-evoked calcium deregulation followed by oxidative-nitrosative stress may significantly affect dopaminergic cells viability. We observed that pre-treatment with antioxidant or NOS inhibitor prevents toxicity evoked by ASN oligomers (Fig. 7a). Additionally, morphological examination of cell nuclei, stained with DNA-binding fluorochrome Hoechst 33342, showed that cells exposed to ASN oligomers presented typical apoptotic morphology, including condensation of chromatin and nuclear fragmentation (Fig. 7b, c). NOS inhibition as well as antioxidant pre-treatment prevented apoptotic cell death caused by extracellular ASN (Fig. 7b, c). To determine whether parkin deregulation is involved in cell death induced by exogenous ASN oligomers, we silenced endogenous parkin with siRNA (Supplementary Fig. S1). The 80% decrease in parkin expression in PC12 cells significantly reduced their viability, but this effect was less profound when compared to control PC12 cells treated with ASN (Fig. 8a). Notably, parkin silencing enhanced the toxicity of exogenous ASN (Fig. 8a). To determine the cytoprotective role of parkin against ASN-induced cell death, PC12 cells were transfected with human parkin gene, which resulted in sixfold increase in parkin protein levels when compared to control cells transfected with the empty vector (Supplementary Fig. S2). We observed that parkin overexpression significantly alleviated ASN-induced cell death when compared with empty vector-transfected cells, suggesting an important role of parkin against ASN-induced neurotoxicity (Fig. 8b).

## Discussion

Although both ASN and parkin mutations and associated dysfunctions are linked to the molecular pathogenesis of PD, few studies investigated the functional interaction between those two proteins. In particular, the potential role of extracellular ASN oligomers on the deregulation of parkin in PD pathology has not been reported. In the present study, we show for the first time that nitrosative stress induced by exogenous ASN oligomers promotes parkin deregulation via *S*-nitrosylation followed by its degradation. Moreover, we demonstrate that parkin deregulation induced by ASN is partially responsible for the toxic effect of ASN in dopaminergic neural cells. Previous studies showed that various oxidative or nitrosative stress conditions can cause posttranslational modifications on parkin (sulfonation and *S*-nitrosylation, respectively) [10, 38]. Moreover, various PD-related toxins were shown to induce alterations in parkin solubility, activity, and intracellular aggregation, resulting in increasing cell death, both in cell culture and in vivo [12, 38, 39]. It was also found that in the presence of various stressors, parkin accumulation into the insoluble fraction of neurons was promoted by endogenous ASN [39]. Our findings are thus consistent with previous studies in demonstrating the functional link between ASN and parkin *S*-nitrosylation in neuronal cells and extend earlier findings by showing that extracellular ASN may be a direct cause for parkin posttranslational modifications in neuronal cells.

Many studies indicated deregulation in calcium homeostasis and elevated release of nitric oxide as important mediators of toxicity induced by extracellular ASN [25]. Recently, it was demonstrated that the negative impact of aberrantly secreted ASN does not appear to involve internalization of this protein by the recipient neurons [40, 41], but it depends on deregulation of various plasma membrane receptors most of which are Ca^2+^ channels [28, 42, 43]. Alternatively, aberrant oligomeric structures of ASN were shown to evoke permeabilization of cellular membranes leading to uncontrolled Ca^2+^ influx and synaptic vesicular depletion [27, 44–47]. In our studies, we generated an oligomeric species of ASN that are able to form annular pore-like structures and observed that treatment of PC12 cells with those oligomers leads to significant increase in cellular Ca^2+^ concentration. Previous studies indicated that elevated calcium is responsible for ASN-induced activation of neuronal nitric oxide synthase (nNOS) and increased NO level [42]. In accordance with those data, our results showed that treatment with exogenous ASN results in elevated NO as well as other free radical production. Under specific conditions, such as a low level of arginine or co-factors, NOS could additionally produce superoxide (O_2_^−^) or hydrogen peroxide (H_2_O_2_). Since in our study exogenous ASN induces significant decrease in arginine level, probably due to both NOS and arginase activation, this may create the favorable circumstances for free radical production and constitute one of the mechanisms of ASN-induced oxidative stress in neurons. Previous data showed that exogenous ASN is able to increase free radical levels by reducing mitochondrial complex I activity [48–50]. Alternatively, direct release of H_2_O_2_ and, subsequently, its conversion into hydroxyl radicals also occur during formation of ASN oligomers [51–53]. The simultaneous production of NO and O_2_^−^ in oxidative stress conditions may lead to the formation of peroxynitrite (ONOO^−^), which is a highly reactive free radical that causes modification of the macromolecules [54]. Although we observed the increasing antioxidative enzymes’ expression, probably as a compensatory mechanism to the enhancement of ROS level, they are unable to readily detoxify the reactive intermediates and restore cellular redox homeostasis disrupted by ASN treatment. These results are with the agreement with previous data showing that accumulation of ASN in cells induces mitochondrial damage and disturbance in the balance between pro- and antioxidative mechanisms [6, 25, 42, 55].

Multiple lines of evidence indicate that oxidative and nitrosative stress could be one of the main causes of neuropathological changes observed in PD [Bhat et al., 2015; Wilkaniec et al., 2013; Dias et al., 2013]. Moreover, nitration or *S*-nitrosylation represents a probable mechanism contributing to the NO-induced misfolding of various proteins, including ASN (Gu et al. 2002; Cho et al. 2009; Nakamura and Lipton 2009). Previous in vitro reports indicated parkin *S*-nitrosylation after treatment with NO donors [9, 10], followed by observation of *S*-nitrosylated parkin in animal models of PD, as well as in human brains with sporadic PD and diffuse Lewy body disease [9, 10, 56]. In line with those data, our results showed that extracellular ASN, through activation of nitrosative stress, induces parkin *S*-nitrosylation and activation leading to decrease in parkin protein level. This is in agreement with previous study by Ozawa et al. [57], showing that endogenous *S*-nitrosylation of parkin is responsible for activation of its E3 ligase activity. Conversely, Dawson’s group claimed that *S*-nitrosylation of parkin is responsible for its inhibition [9], whereas Yao et al. [10] showed that NO-mediated posttranslational modifications initially lead to a dramatic increase in parkin activity followed by a decrease in the E3 ligase-ubiquitin-proteasome degradative pathway. The possible reasons of discrepancies between results observed by these three groups were largely discussed in Ozawa’s paper, suggesting that regulation of parkin ligase activity by *S*-nitrosylation/denitrosylation might be time-dependent and that actually denitrosylation might be the cause of the reduced E3 ligase activity of parkin observed at later time points.

Interestingly, in our study, we observed that the level of *S*-nitrosylated parkin was greatly increased 24 h after ASN treatment and corresponded to the increase in parkin activity, while longer 48-h treatment normalized parkin activity to the basal level. These differences between our data and the results obtained by others might depend on the duration of NO release in cells. In previous papers, different NO donors or complex I inhibitors as well as the mitochondrial-uncoupling reagents induced fast increase, followed by significant decrease in NO liberation [57]. This resulted in parkin *S*-nitrosylation up to 3 h of treatment followed by denitrosylation at time points when the NO level was no longer increased. In accordance with our previous data showing the progressive long-term rise of in the cytosolic Ca^2+^ in ASN-treated cells [33], we observed in this study that extracellular ASN induces long-lasting increase in NO level. Apart from deregulating NOS activity, extracellular ASN was demonstrated to exert its sustained action by elevation of nNOS expression [58]. Thus, we suggest that the long-lasting elevation of NO level induced by extracellular ASN might result in prolonged parkin *S*-nitrosylation and activation. It is noteworthy that at the same time with the elevated autoubiquitination of parkin, the level of total ubiquitin did not change substantially after ASN treatment, suggesting that even though *S*-nitrosylation activates parkin, this enzyme is, at least in part, ineffective in marking its substrates for degradation. One possible explanation of this phenomenon is that while extracellular ASN increased the autoubiquitination of parkin through *S*-nitrosylation, at the same time, it decreased parkin protein levels, which resulted in dampening of the parkin E3 ubiquitin ligase activity. It was also concluded that autoubiquitination of parkin upon its *S*-nitrosylation may be directly responsible for inhibiting its E3 ligase activity, resulting in impaired ubiquitination and degradation of neurotoxic proteins, thereby contributing to their accumulation/aggregation resulting in ER stress and neuronal cell injury or death [59].

Finally, we found that either restoration of parkin homeostasis in PC12 cells by blocking its *S*-nitrosylation or elevation of parkin expression levels exerted cytoprotective effects against ASN oligomer toxicity. These findings are in agreement with currently emerging evidence indicating parkin as an important neuroprotective protein [60–62]. Previous reports demonstrated that E3 ubiquitin ligase activity of parkin was involved in many mechanisms regulating cell viability, such as, for example, governing mitochondrial quality control by triggering selective mitophagy [63] or participation in protein degradation during ER stress [64]. Thus, *S*-nitrosylation of parkin resulting in the inhibition of its ubiquitin E3 ligase activity may significantly contribute to the neurodegeneration by impairing these cytoprotective mechanisms [59].

Of note, the deleterious effects of ASN may not be completely dependent on deregulation of parkin, as silencing of parkin expression in PC12 cells induced already significant cell loss. This effect on cell viability, however, was less profound compared to the cytotoxicity evoked by ASN oligomers. It is in agreement with previous data showing that ASN is able to directly translocate to the mitochondria and disturb the Complex I function leading to neurodegeneration [65, 66]. Moreover, the cellular accumulation of ASN was demonstrated to induce nitrosylation of many mitochondrial proteins [55], leading to dysfunction of these organelles followed by cytochrome c release, caspase-3 activation, and p53-independent cell death [6, 67]. Nevertheless, we observed that in the conditions when parkin is almost completely down-regulated the toxic effects of ASN are significantly augmented, while parkin overexpression protects against ASN-evoked cell death. In agreement with these observations in the present study, it was demonstrated that parkin deficiency could be a trigger of oxidative stress and mitochondrial impairment [68] due to decreased cellular antioxidant defense, i.e., reduced glutathione levels and declining SOD activity [69]. On the other hand, parkin overexpression has been found to prevent neuronal degeneration induced by both mutant and wild-type ASN in primary neurons or neuroblastoma cells [70, 71]. This suggests that parkin can protect neuronal cells against the ASN toxicity, but in the conditions of exacerbated oxidative/nitrosative stress, this protective effect may be abrogated by *S*-nitrosylation of parkin leading to its degradation.

In summary, our data is the first to show that ASN-dependent induction of oxidative/nitrosative stress is a key molecular process involved in parkin *S*-nitrosylation leading to dysregulation of its activity and degradation. Those new findings provide compelling evidence for direct association of parkin dysfunction to extracellular ASN-signaling as a critical phenomenon occurring in pathophysiology of sporadic PD. Thus, inhibiting parkin *S*-nitrosylation or elevating its level may contribute to the development of new therapies for PD and other synucleinopathies.

## Electronic supplementary material

S.I. Fig. 1 Parkin silencing in PC12 cells. **A)** Expression of *Park2* gene in PC12 cells after 24 h treatment with 20–60 nM Parkin siRNA measured by RT-PCR. Data represent the mean value ± S.E.M. for 4 independent experiments. Results were normalized to GAPDH level. *p < 0.05; **p < 0.01 compared to corresponding control siRNA, using Student’s *t*-test. **B)** Parkin immunoreactivity in PC12 cells after 24 h treatment with 20–60 nM Parkin siRNA measured by Western blot. Results were normalized to actin level. Data represent the mean value ± S.E.M. for 5 independent experiments. ***p < 0.001 compared to corresponding control siRNA, using Student’s *t*-test (GIF 190 kb)

High Resolution Image (TIF 4337 kb)

S.I. Fig. 2 Parkin overexpression in PC12 cells. **A)** Restriction analysis of obtained clones pcDNA3.4+*PARK2*. The plasmid DNA for each clone was isolated from the bacteria and then digested with restriction enzymes AscI and PacI and electrophoretically separated on a 1% agarose gel. Gel was stained with ethidium bromide and visualized on a transilluminator. **B)** Parkin immunoreactivity in Park2 overexpressed PC12 cells measured by Western blot. Results were normalized to Gapdh level. Data represent the mean value ± S.E.M. for 5 independent experiments. ***p < 0.001 compared to control, using Student’s *t*-test (GIF 91 kb)

High Resolution Image (TIF 1965 kb)
