# Supplementary figures and images for: Extracellular Alpha-Synuclein Oligomers Induce Parkin S-Nitrosylation: Relevance to Sporadic Parkinson’s Disease Etiopathology
Source: Mol Neurobiol. 2018 Apr 21;56(1):125–40. doi: 10.1007/s12035-018-1082-0 (PMC6334739; doi:10.1007/s12035-018-1082-0)

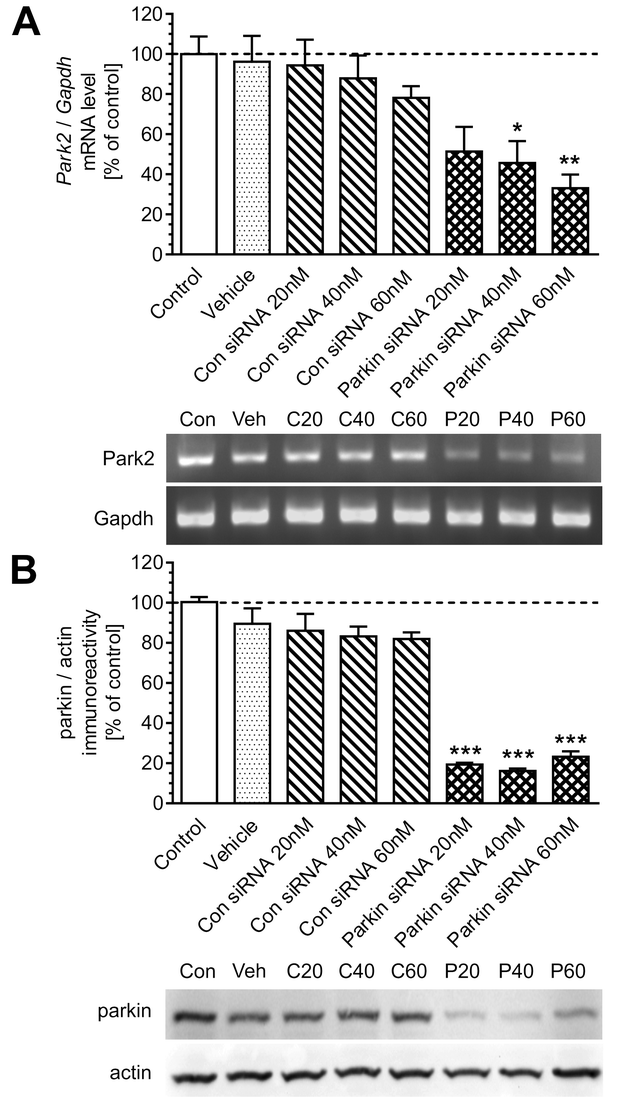

Supplement: Supplementary file 1 — Parkin silencing in PC12 cells. A) Expression of Park2 gene in PC12 cells after 24 h treatment with 20–60 nM Parkin siRNA measured by RT-PCR. Data represent the mean value ± S.E.M. for 4 independent experiments. Results were normalized to GAPDH level. *p < 0.05; **p < 0.01 compared to corresponding control siRNA, using Student’s t-test. B) Parkin immunoreactivity in PC12 cells after 24 h treatment with 20–60 nM Parkin siRNA measured by Western blot. Results were normalized to actin level. Data represent the mean value ± S.E.M. for 5 independent experiments. ***p < 0.001 compared to corresponding control siRNA, using Student’s t-test (GIF 190 kb) [file 12035_2018_1082_Fig9_ESM.gif]

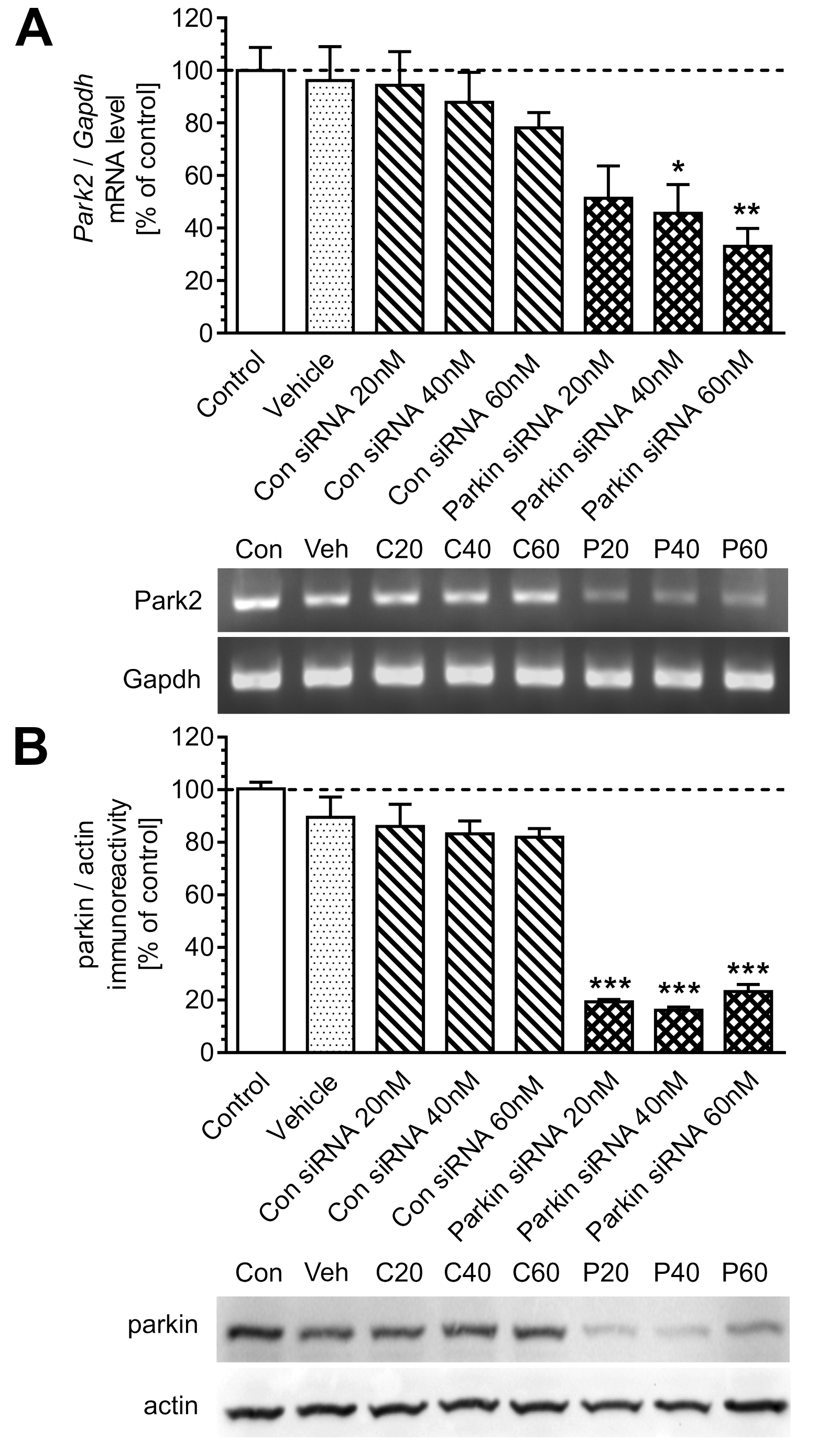

Supplement: Supplementary file 2 — High Resolution Image (TIF 4337 kb) [file 12035_2018_1082_MOESM1_ESM.tif]

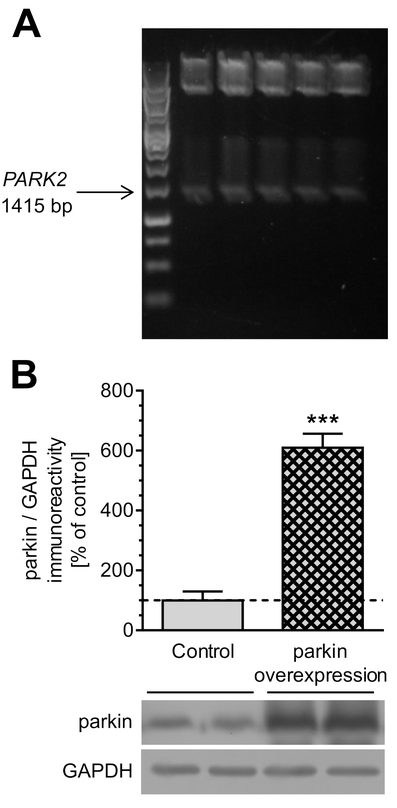

Supplement: Supplementary file 3 — Parkin overexpression in PC12 cells. A) Restriction analysis of obtained clones pcDNA3.4+PARK2. The plasmid DNA for each clone was isolated from the bacteria and then digested with restriction enzymes AscI and PacI and electrophoretically separated on a 1% agarose gel. Gel was stained with ethidium bromide and visualized on a transilluminator. B) Parkin immunoreactivity in Park2 overexpressed PC12 cells measured by Western blot. Results were normalized to Gapdh level. Data represent the mean value ± S.E.M. for 5 independent experiments. ***p < 0.001 compared to control, using Student’s t-test (GIF 91 kb) [file 12035_2018_1082_Fig10_ESM.gif]

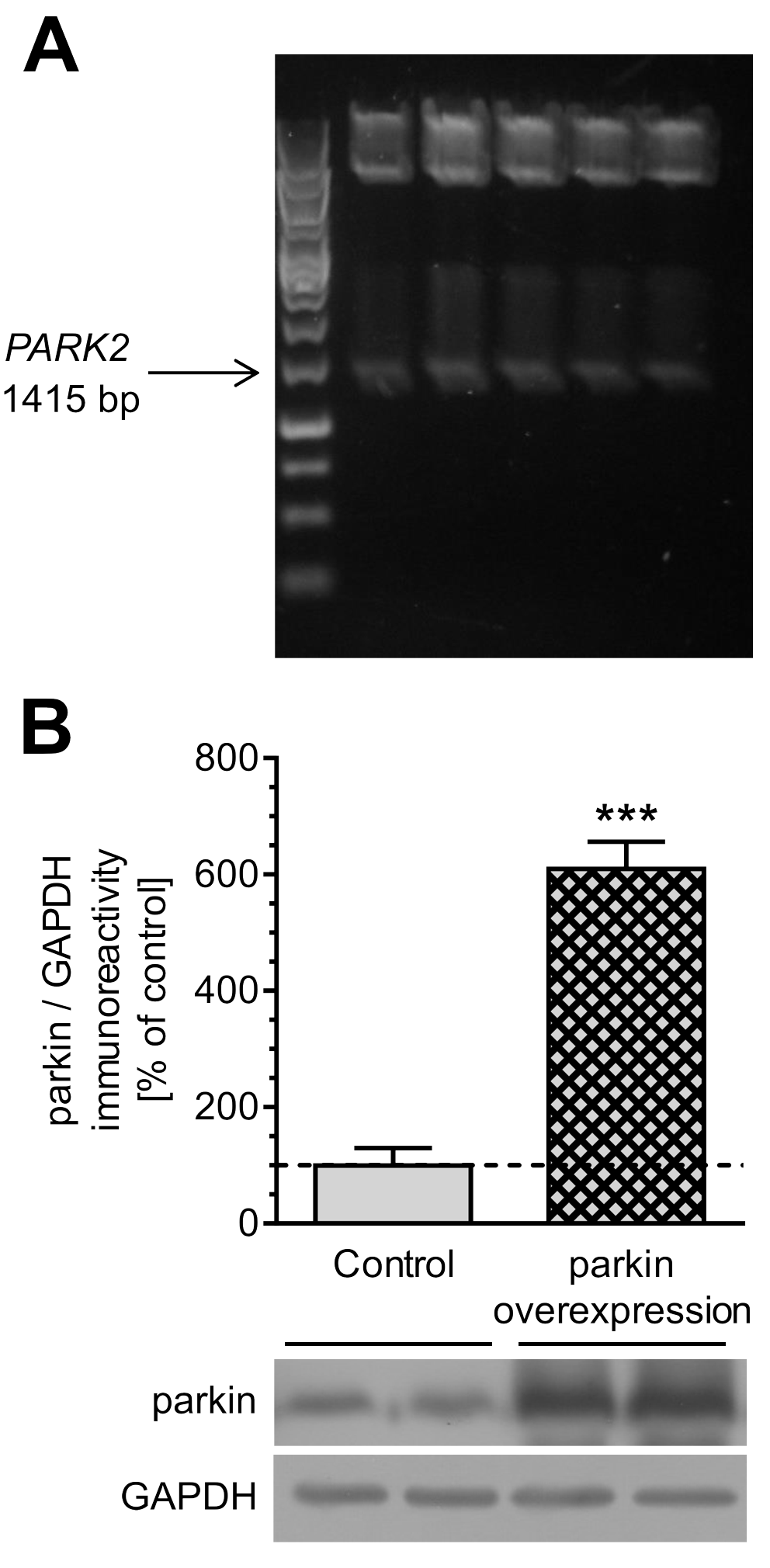

Supplement: Supplementary file 4 — High Resolution Image (TIF 1965 kb) [file 12035_2018_1082_MOESM2_ESM.tif]
